# Progress in Medicine

**Published:** 1899-08-05

**Authors:** 


### Progress in Medicine.

### DISEASES OF THE BLOOD.

**Splenic Leucæmia.**—Lauder Brunton[1] discusses a case in a boy, aged 12½, marked by hæmorrhage from the mouth, abdominal pain, and feverish attacks, together with a tumour formed by a greatly enlarged spleen. In the diagnosis of a large spleen he lays great stress upon the direction of the notch, which should point upwards and to the right. By neglecting this point a malignant mass has been mistaken for a spleen. Another important matter is to avoid inserting a needle, for these spleens may bleed profusely from a small puncture. . . . The treatment of the disease itself is not hopeful, and Brunton's experience of arsenic is not more satisfactory than that of other authors. He advises, in addition, iron, quinine, and cod-liver oil, with gentle massage over the spleen and the faradic current, but improvement is, as a rule, only temporary. According to Pye Smith, Hodgkin's disease may include leucæmia, for in the latter disease there are generally other glands enlarged besides the spleen. There are, in fact, cases when the spleen is of normal size, though the lymphatic glands generally are enlarged. He regards a dark red reaction of the urine when heated with H.Cl. as indicative either of a severe anæmia or of malignant disease of the stomach. Others hold that it occurs in ordinary constipation. It is worth noting that marked anæmia in males is not infrequently due to continued hæmorrhage from piles, and the possibility of this cause should not be overlooked in the examination of a case.

James Crook[2] draws attention to the headaches, priapisms, enlarged spleen, and irregular hæmorrhages which occur among the early symptoms of leucæmia splenica. The other variety, the lymphatic, is rare and more rapid in its course, lasting usually about four months, instead of as many years. The presence of nucleated red corpuscles is a special symptom in splenic leucæmia, as well as the increase of white cells, particularly of the mononuclear ones and myelocytes. The latter are hardly ever seen elsewhere—not even in the lymphatic form of the disease, where the chief change is the increase of the lymphocytes. With the enlargement of the spleen some increase in size of the liver is generally noted, especially in the later stages. In the discussion upon this paper Weltzmiller referred to the difficulty of getting red marrow from finely-crushed bone. He would give this in a concentrated form, as well as iron and arsenic combined. In a case of the lymphatic type, where, however, the liver and spleen, as well as the small glands, were enlarged, Wenzel[3] notices that the patient died from asphyxia due to compression of the trachea and pulmonary artery by enlarged glands.

**Acute Leucæmia.**—Two cases are reported by Fussell, Jobson, and Taylor,[4] who analysed 54 others collected from various sources. The most striking symptom is the rapid course, which lasted in these patients on the average only 39 days. It is usually found in early life, most of the cases occurring in persons between 10 and 30 years of age. The leucocytosis is moderate, and the red cells often fall below 2,000,000 per c.m.m. The greatest increase is in the lymphocytes. Usually the polynuclear ones are normal, and eosinophiles few. Occasionally myelocytes are present. Besides hæmorrhages and fever there is an enlargement of the lymph glands, and to some extent of the spleen and liver. All the cases collected terminated fatally.

In a case of splenic leucæmia in a young woman, J. Finlayson[5] records the occurrence of retinal and conjunctival hæmorrhages, and also of others in the internal ear, notably the vestibule and the first turn of the cochlea. These were ushered in by tinnitus, nausea, and vertigo, with sudden deafness. The Eustachian tubes were easily inflated without improvement of the deafness. A watch could not be heard in contact with the ears, and the tuning-fork was heard longer by air conduction than by bone. It may be added that the spleen weighed 6 lb. 4 oz.

[1] Treatment, April. [2] The Post Graduate, Jan. [3] Brit. Med. Jour., Feb. 11. [4] Med. Chron., Feb. [5] Brit. Med. Jour., Dec. 31.

